# Muscle Adaptations to Heavy-Load and Blood Flow Restriction Resistance Training Methods

**DOI:** 10.3389/fphys.2022.837697

**Published:** 2022-02-03

**Authors:** Anthony K. May, Aaron P. Russell, Paul A. Della Gatta, Stuart A. Warmington

**Affiliations:** Institute for Physical Activity and Nutrition, School of Exercise and Nutrition Sciences, Deakin University, Geelong, VIC, Australia

**Keywords:** hypertrophy, strength, vascular occlusion, skeletal muscle, growth markers

## Abstract

Resistance-based blood flow restriction training (BFRT) improves skeletal muscle strength and size. Unlike heavy-load resistance training (HLRT), there is debate as to whether strength adaptations following BFRT interventions can be primarily attributed to concurrent muscle hypertrophy, as the magnitude of hypertrophy is often minor. The present study aimed to investigate the effect of 7 weeks of BFRT and HLRT on muscle strength and hypertrophy. The expression of protein growth markers from muscle biopsy samples was also measured. Male participants were allocated to moderately heavy-load training (HL; *n* = 9), low-load BFRT (LL + BFR; *n* = 8), or a control (CON; *n* = 9) group to control for the effect of time. HL and LL + BFR completed 21 training sessions (3 d.week^−1^) comprising bilateral knee extension and knee flexion exercises (HL = 70% one-repetition maximum (1-RM), LL + BFR = 20% 1-RM + blood flow restriction). Bilateral knee extension and flexion 1-RM strength were assessed, and leg muscle CSA was measured *via* peripheral quantitative computed tomography. Protein growth markers were measured in vastus lateralis biopsy samples taken pre- and post the first and last training sessions. Biopsy samples were also taken from CON at the same time intervals as HL and LL + BFR. Knee extension 1-RM strength increased in HL (19%) and LL + BFR (19%) but not CON (2%; *p* < 0.05). Knee flexion 1-RM strength increased similarly between all groups, as did muscle CSA (50% femur length; HL = 2.2%, LL + BFR = 3.0%, CON = 2.1%; TIME main effects). 4E-BP1 (Thr37/46) phosphorylation was lower in HL and LL + BFR immediately post-exercise compared with CON in both sessions (*p* < 0.05). Expression of other growth markers was similar between groups (*p* > 0.05). Overall, BFRT and HLRT improved muscle strength and size similarly, with comparable changes in intramuscular protein growth marker expression, both acutely and chronically, suggesting the activation of similar anabolic pathways. However, the low magnitude of muscle hypertrophy was not significantly different to the non-training control suggesting that strength adaptation following 7 weeks of BFRT is not driven by hypertrophy, but rather neurological adaptation.

## Introduction

Blood flow restriction training (BFRT) generally comprises periods of low-intensity resistance or aerobic exercise with blood flow restriction (BFR) applied to the working limbs *via* pneumatic or elastic cuffs. Resistance-based BFRT can increase skeletal muscle strength and induce muscle hypertrophy to a greater degree than equal-intensity training without the application of BFR (Kubo et al., 2006; Takada et al., 2012). Such muscle adaptation without utilizing large mechanical loads has positioned BFRT as a potential alternative or complementary training method to heavy-load resistance training (HLRT), particularly for lower physical functioning populations, such as those undergoing musculoskeletal rehabilitation or the frail elderly.

BFRT utilizes lighter external loads than HLRT (BFRT = 20–30% one-repetition maximum (1-RM); HLRT >60% 1-RM), induces reduced-to-similar adaptations to muscle strength (Lixandrão et al., 2018; Grønfeldt et al., 2020), and comparable hypertrophy (Lixandrão et al., 2018). Enhanced muscle force generating capacity following HLRT is largely due to neuromuscular adaptation, rather than hypertrophy (Kraemer et al., 1996). Indeed, the causative role of hypertrophy in resistance training-based strength adaptations in humans is still questioned (Dankel et al., 2018). However, the contributions of hypertrophy vs. neuromuscular adaptation to strength gains following BFRT are less clear. BFRT induces muscle hypertrophy within 2 weeks (Abe et al., 2006; Hill et al., 2018) and can induce hypertrophy without changes to neural activation (Kubo et al., 2006). These findings, combined with some observations of similar percentage changes in muscle strength and hypertrophy (Kubo et al., 2006; Martín-Hernández et al., 2013; Ozaki et al., 2013), suggest that hypertrophy may be a major factor contributing to BFRT-induced strength gains. In contrast, pronounced BFRT-induced strength adaptations may be incongruent with minor/no muscle hypertrophy (Laurentino et al., 2012; Lixandrão et al., 2015; Vechin et al., 2015; Cook et al., 2018). For example, BFRT and HLRT increased leg extension 1-RM strength similarly (23.5% average) when compared to an untrained control group (Cook et al., 2018), but meaningful muscle hypertrophy did not occur as the change in muscle volume (4.5%) was not different to an untrained control group. This suggests that if hypertrophic adaptation is induced following BFRT, it may have minimal contribution to strength change; the latter most likely driven by neurological adaptations (Jessee et al., 2018).

Mechanical tension is the dominant primary stimulus for anabolic activity during HLRT (Pearson and Hussain, 2015). Comparatively, BFRT produces less mechanical tension due to lower loads but induces enhanced metabolic stress (Pearson and Hussain, 2015). It is therefore possible that regulation of intramuscular signaling pathways differs between training methods. Protein kinase B (Akt)/mammalian target of rapamycin (mTOR) pathway signaling increases protein synthesis (Rommel et al., 2001; Wang and Proud, 2006) and is responsive to chronic (Léger et al., 2006) and single-bout (acute) HLRT (Dreyer et al., 2006, 2008, 2010), as well as acute BFRT (Fujita et al., 2007; Fry et al., 2010; Gundermann et al., 2012; Wernbom et al., 2013). As such, similar cell signaling events may regulate muscle growth following HLRT and BFRT. Comparatively, anabolic mitogen-activated protein kinase (MAPK) cascade activity in BFRT is somewhat uncharacterized. MAPKs generally activate following traditional resistance exercise (Boppart et al., 1999; Martineau and Gardiner, 2001; Williamson et al., 2003; Deldicque et al., 2008), but extracellular signal-regulated kinase (ERK) 1/2 activation has been unchanged following BFRT (Gundermann et al., 2012, 2014). In addition, c-Jun N-terminal kinase (JNK) appears sensitive to mechanical tension and muscle damage (Aronson et al., 1997), which appear lower in BFRT, though has not been investigated. Furthermore, the acute response to BFRT following a training program (i.e., the chronic effect of training) is yet to be investigated. Activation of key translation initiation proteins appears preserved in young adult muscle following traditional resistance training (Farnfield et al., 2012), but it is unclear if this is also the case following BFRT.

This study aimed to compare the effect of 7 weeks of BFRT and HLRT on changes in muscle strength and size to investigate the role of muscle hypertrophy in BFRT-induced strength adaptation. A secondary aim was to investigate the mechanisms of BFRT-induced hypertrophy *via* intracellular signaling proteins that affect muscle growth by confirming the role of Akt, and exploring the role of other potential pathways, both acutely and chronically.

## Materials and Methods

### Participants

Twenty-six untrained young men were recruited for this study. Participants were matched for bilateral leg extension 1-RM strength then randomly allocated to either a low-load resistance BFRT group (LL + BFR; *n* = 8), a moderately heavy-load resistance training group without BFR (HL; *n* = 9), or a passive non-training control group (CON; *n* = 9). All participants had not undertaken any regular resistance exercise within the previous 6 months and did not present with any musculoskeletal, neurological, or vascular disease/injury. Prior to inclusion in the study, all participants provided written informed consent and underwent a pre-screening procedure including a health questionnaire. Participants were excluded if presenting with diagnosed diabetes mellitus, hypertension, or if taking medication prescribed for blood pressure control. In addition, participants at increased risk of complications due to muscle biopsies, such as those with blood clotting disorders or prescribed with blood thinning medications, were also excluded. This study was carried out in accordance with the recommendations of the Deakin University Human Ethics Advisory Group. All participants gave written informed consent in accordance with the Declaration of Helsinki. The protocol was approved by the Deakin University Human Ethics Advisory Group (Project number 2014-229).

### Experimental Design

**Figure 1:**
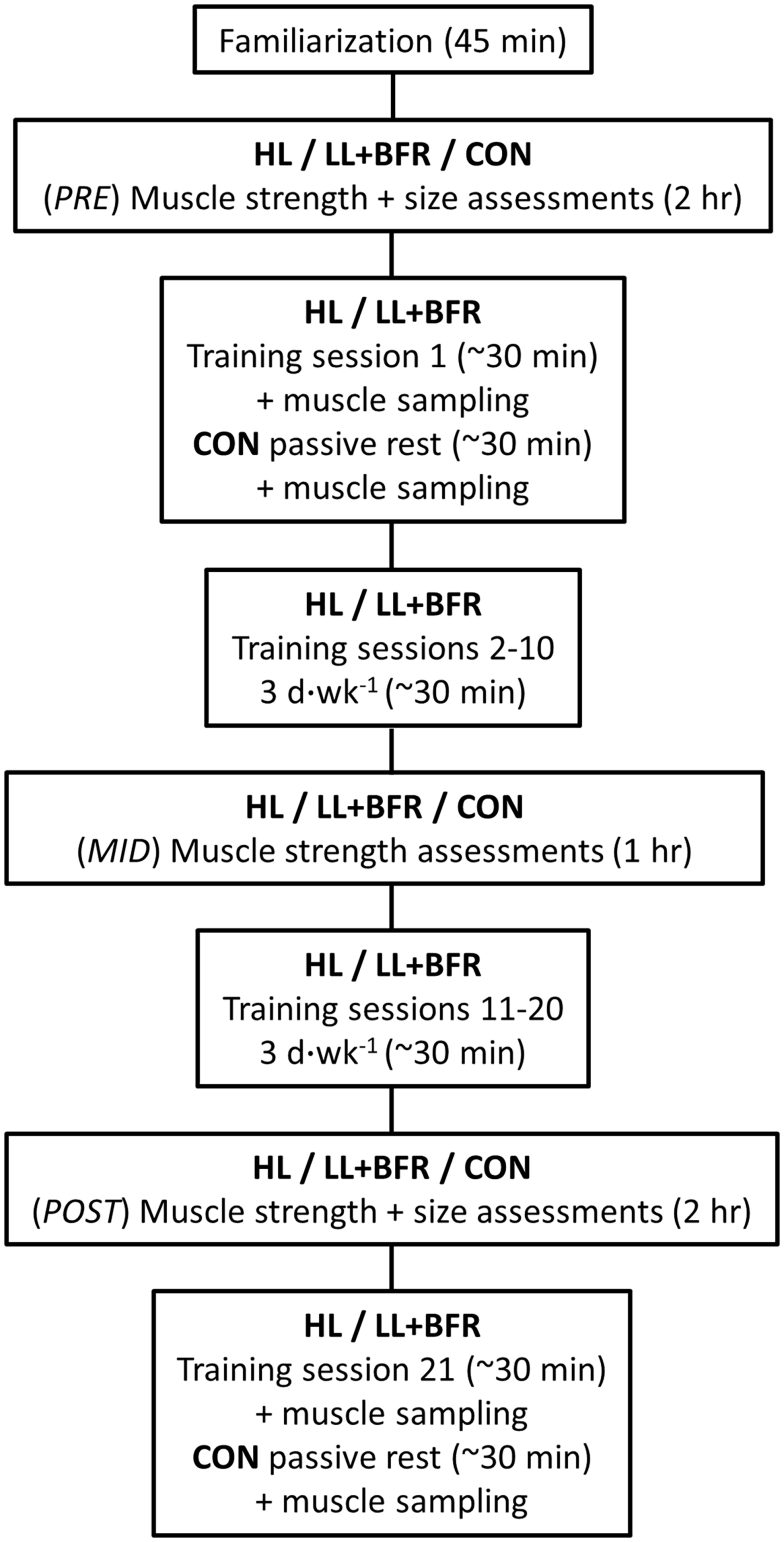
Experimental protocol. HL, heavy-load resistance training group; LL + BFR, blood flow restriction training group; and CON, non-training control group.

The overall experimental design is displayed in Figure 1. All participants first completed a familiarization session, followed by a testing session at least 3 days later (PRE). PRE included a muscle cross-sectional area (CSA) measurement of the dominant leg, followed by 1-RM strength assessments of the lower body. One week following PRE, HL and LL + BFR performed 20 training sessions (3 d.week^−1^) over 7 weeks. Training sessions comprised bilateral knee extension and knee flexion exercises. During training, LL + BFR had blood flow restricted by pressurized cuffs. The initial session (Session 1) of the training program, or passive rest in the case of CON, included an excision of muscle *via* percutaneous muscle biopsies for growth marker protein analysis. Attendance from CON was not required for any other session in the training block. Muscle strength was assessed following completion of 10 training sessions (MID), and both muscle strength and size were assessed on a final occasion 3–5 days following completion of the last (20th) training session (POST). One week following the final muscle size and strength assessment, all participant groups attended the laboratory for a final exercise session (for HL and LL + BFR) involving muscle sampling (Session 21). A single researcher performed all testing and biochemical analyses and was not blinded to participant grouping allocations.

### Familiarization

After an initial pre-screening process, participants were familiarized with the laboratory and machinery used throughout the program. Participants were then instructed on exercises used within the study, which was followed by an initial familiarization with BFRT that comprised light bilateral dumbbell bicep curl exercises where the dominant arm had BFR applied and the non-dominant arm did not. This was performed to inform participants of the unique sensations expected during BFRT. This BFR familiarization was not performed in the legs, which would be trained with BFR applied if the participant were later allocated to LL + BFR, to minimize any influences on lifting performance in future.

### Training Protocol

Allocated exercise loads in the first 10 training sessions were calculated as a percentage of 1-RM measured during PRE strength testing and sessions 11–20 from the MID strength testing. In addition, loads for Session 21 were calculated using results from POST.

Training sessions began with a general 5-min warm-up on a cycle ergometer. Participants in HL then performed four sets of knee flexion and knee extension exercises at a repetition velocity of 2 s eccentric, 2 s concentric phase, guided by a metronome. Exercises were performed at 70% 1-RM without BFR applied and comprised eight repetitions each set. Rest periods were 2 min each as per recommendations for muscle strength and hypertrophy adaptations (American College of Sports Medicine, 2009). Participants in LL + BFR performed four sets of lower-body exercises at 20% 1-RM with BFR applied to the most proximal portion of the upper legs. Training sessions comprised a set of 30 repetitions, followed by three sets of 15 repetitions. 30-s rest periods with continuous cuff inflation were utilized as is standard practice for BFRT to increase metabolic stress (Scott et al., 2015). Repetition velocity matched that of HL. The order of knee flexion/extension exercises alternated every training session. CON did not perform any resistance training during the training period but continued their daily habits. During study participation, all groups were not permitted to perform any structured resistive exercise outside of laboratory sessions.

### Muscle Strength

Bilateral knee flexion and knee extension strength were measured *via* 1-RM assessments utilizing pneumatic resistance machinery; a seated bilateral knee extension machine (Keiser Air200 leg extension, Keiser Corporation, Fresno, United States) and a prone knee flexion machine (Keiser Air200 leg curl, Keiser Corporation, Fresno, United States). The same machinery was also utilized for training sessions for HL and LL + BFR.

All 1-RM strength testing followed the American College of Sports Medicine (ACSM) guidelines for maximal muscle strength assessment (Thompson et al., 2010). 1-RM tests were preceded by a general 5-min warm-up on a cycle ergometer, then a specific resistance submaximal warm-up for both following 1-RM exercises. Successful attempts required the participant to lift the resistance through a full range of motion with control. Participants were permitted to grip the handles of the machines in training and testing sessions. Fixation belts were not utilized.

### Muscle Cross-Sectional Area

Muscle CSA of the dominant leg was measured at PRE and POST *via* peripheral quantitative computed tomography (pQCT; Stratec XCT3000, Stratec Medizintechnik, Baden-Württemberg, Germany). Scans were performed at 25 and 50% femur length in the dominant leg (Seynnes et al., 2007). All measurements were performed by the same researcher with the participant laying supine and the scanned limb positioned through the center of the pQCT gantry. Participants were asked to remain still and to breathe normally during scans, which were acquired with a voxel size of 0.5 mm. pQCT images were analyzed using the BoneJ plugin for ImageJ (Doube et al., 2010). Muscle tissue was defined as voxels with a density >40 and <200 mg/cm^2^ (Rantalainen et al., 2014); then, total muscle CSA was calculated for all soft tissue within those thresholds. CSA of grouped knee flexor and extensor muscles were also independently analyzed at 25% of femur length but not 50% femur length due to unclear separation of muscles at those sites with the resolution available.

### Blood Flow Restriction

LL + BFR performed the training protocol with blood flow to the lower-body restricted using cuffs (86 cm length, 10.5 cm width, 8 cm bladder width, nylon material) attached to an automatic tourniquet system (Zimmer ATS 4000, Zimmer Biomet, Warsaw, United States). BFR cuffs were applied to the most proximal portion of both upper legs and inflated immediately prior to commencement of the first set of exercises (either knee flexion or extension). Cuffs remained inflated continuously throughout all training sessions until completion of the final set of subsequent lower-body exercises (roughly 14-min inflation per session).

On arriving at the laboratory for Session 1, prior to administration of anesthetic, individualized limb occlusion pressure (LOP) of the lower limbs, the pressure required to completely occlude peripheral tissue blood flow, was assessed (Table 1) using previously reported methods (May et al., 2018). The BFR restriction pressure for the following 10 training sessions was set to 60% LOP. Assessment of LOP was also repeated prior to commencement of the 11th training session (Session 11) and 60% LOP was utilized as the restriction pressure for all remaining sessions.

**Table 1:** Participant anthropometry, and limb occlusion pressure (LOP) in heavy-load resistance training (HL), blood flow restriction training (LL + BFR), and non-training control (CON) groups.

Variable	HL	LL + BFR	CON
Age (years)	24.1 ± 3.8	24.1 ± 4.0	21.2 ± 2.2
Height (cm)	179.8 ± 8.7	185.3 ± 9.4	181.3 ± 4.3
Session 1 Body mass (kg)	78.8 ± 16.7	85.3 ± 16.2	85.1 ± 23.2
Session 21 Body mass (kg)	79.4 ± 16.2	85.2 ± 16.2	86.7 ± 24.3^*^
Session 1 BMI (kg∙m^−2^)	24.3 ± 4.4	24.7 ± 3.8	25.7 ± 6.2
Session 21 BMI (kg∙m^−2^)	24.5 ± 4.3	24.7 ± 3.8	26.2 ± 6.5^*^
Session 1 LOP (mmHg)	-	215 ± 19	-
Training session 1–10 restriction pressure (60% LOP; mmHg)	-	129 ± 11	-
Session 11 LOP (mmHg)	-	212 ± 17	-
Training session 11–20 restriction pressure (60% LOP; mmHg)	-	127 ± 10	-

Data are mean ± SD. BMI, body mass index; BFR, blood flow restriction.

* Different from Session 1 (*p* < 0.05).

### Muscle Biopsy Sessions

**Figure 2:**
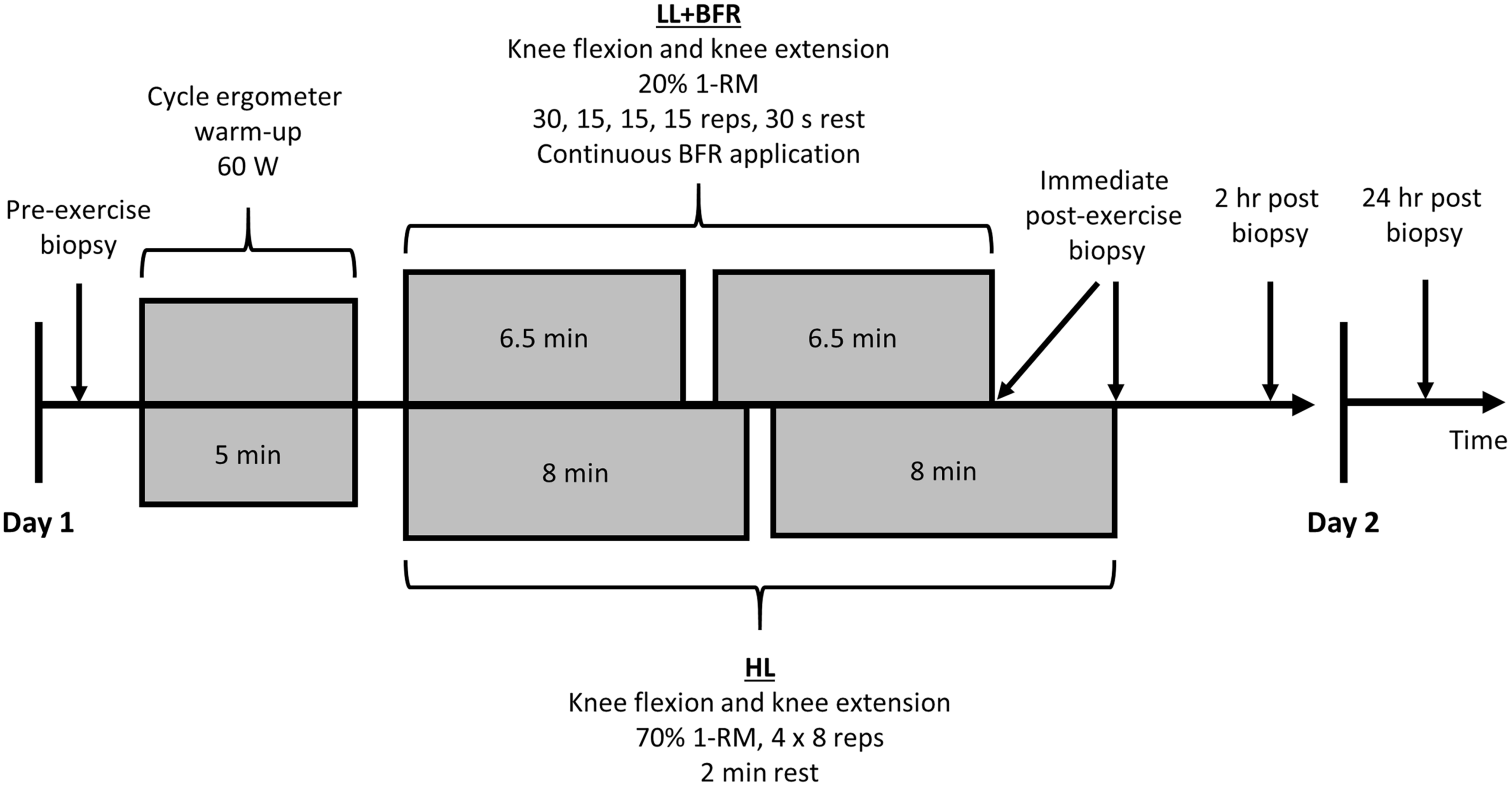
Skeletal muscle sampling sessions for heavy-load resistance training (HL) and blood flow restriction training (LL + BFR) groups. A non-training control group (CON) also performed 30 min passive rest between the first and second biopsies. 1-RM, one-repetition maximum and BFR, blood flow restriction.

Skeletal muscle sampling *via* muscle biopsies occurred during Session 1 and Session 21, both of which required all participants to report to the laboratory on two consecutive days. These sessions each involved four biopsies of the vastus lateralis muscle (Figure 2). Exercise was not permitted on the day of muscle sampling sessions, or the day prior. Participants commenced the sessions at roughly the same time of day (<1-h variation) in a fasted state. Standard dinners were consumed the night before any muscle sampling days. Meals consisted of: energy, 12,090 kJ; protein, 20.2 g; fat (total), 9.6 g; fat (saturated), 4.2 g; carbohydrate (total), 32.0 g; carbohydrate (sugars), 5.4 g; and sodium, 1,060 mg.

Using a Bergström needle (Bergström, 1962), with applied suction (Evans et al., 1982), muscle biopsies were taken from the vastus lateralis of participants under local anesthesia (1% Xylocaine). Generally, 100–200 mg of sample was obtained for each muscle biopsy then immediately frozen in liquid nitrogen and stored at −80°C for protein extraction and analysis. Serial biopsy samples were taken on opposite legs (i.e., left, right, left, and right) and collected at least 2 cm from previous biopsy sites.

After the initial muscle biopsy on Session 1 and Session 21, participants allocated to CON then passively sat or lay supine for 30 min. Participants allocated to HL and LL + BFR immediately began the exercise session (following ~30 s walking to exercise machinery). After completion of a 5-min warm-up on a cycle ergometer at 60 watts, allocated training programs were performed under supervision. Knee extension exercises were performed before knee flexion exercises on both Session 1 and 21. Within 2 min of exercise completion, an immediate post-exercise muscle biopsy occurred. CON also had a sample taken following the passive rest period. All participants then rested passively for 2 h, after which another muscle biopsy occurred. Sampling sites were then covered, and participants were provided with a standard lunch to ensure consistent post-exercise diets. Participants reported back to the laboratory in a fasted state for one final muscle biopsy 24 h following exercise completion or in the case of CON, 24 h following the second muscle biopsy. In total, eight muscle biopsy samples were obtained from all participants over the two sampling sessions.

### Protein Extraction and Western Blots

Total protein from biopsy samples was extracted using RIPA buffer (Merck, Kilsyth, Australia) with 1 μL.ml^−1^ protease inhibitor cocktail (Sigma-Aldrich, Castle Hill, Australia) and 10 μL.ml^−1^ phosphatase inhibitor cocktail (Thermo Fisher Scientific, Scoresby, Australia). Total protein content was determined using a BCA protein assay kit (Thermo Fisher Scientific, Scoresby, Australia).

Equal amounts of protein were separated on 4–15% Criterion™ TGX Stain-Free™ precast gels (Bio-Rad, Gladesville, Australia) in 10% Tris/Glycine/SDS buffer solution. Proteins were then transferred for 30 min into Immobilin-FL PVDF membranes (Millipore, Billerica, United States) and membranes were scanned to quantify total protein transferred in individual wells using a Bio-Rad Gel Doc™ XR+ (Bio-Rad Laboratories, Hercules, United States). Membranes were then blocked for 1 h at room temperature in 5% skim milk powder/10% Tris-buffered saline with 0.1% Tween 20 (TBST).

After blocking, blots were cut and separated, which was followed by overnight incubation with gentle agitation at 4°C in 5% bovine serum albumin/TBST combined with primary antibodies. The following antibodies were purchased from Cell Signaling Technology (Danvers, United States): Phospho-mTOR (Ser2448), Phospho-eukaryotic translation initiation factor 4E-binding protein 1 (4E-BP1; Thr37/46), Phospho-ERK 1/2 (Thr202/Tyr204), Phospho-Stress-activated protein kinase (SAPK)/JNK (Thr183/Tyr185), total mTOR, total ribosomal S6 kinase 1 (S6K1), total 4E-BP1, total ERK 1/2, and total SAPK/JNK. In addition, total muscle RING finger protein-1 (MuRF-1) was purchased from Taylor Bio-Medical (Ashfield, Australia). All antibodies were used in a dilution of 1:1,000 except Total 4E-BP1 (1:500) and Phospho-ERK 1/2 (Thr202/Tyr204; 1:2,000). Only phosphorylation data are shown in this article. All other results are shown in Supplementary Material.

Following overnight incubation, membranes were washed in 10% TBST for 3 × 5-min periods then incubated with gentle agitation for 1 h at room temperature with an appropriate secondary antibody (1:15,000) in a 5% bovine serum albumin/TBST solution. Following incubation, blots were washed again in 10% TBST then exposed on an Odyssey® Infrared Imaging System (LI-COR Biosciences, Lincoln, United States) and individual protein band optical densities were determined using Image Studio Lite (V5.2.5; LI-COR Biosciences, Lincoln, United States). All blots were normalized to the total protein load and an internal control.

### Data Presentation and Statistical Analysis

Power analyses indicated that 24 participants (eight participants per group) were required to detect differences in 1-RM strength, muscle CSA, and acute mTOR (Ser2448) phosphorylation, with 80% power. Thirty participants were recruited and four dropped out citing muscle biopsy concerns (13% attrition). Unless otherwise stated, data are presented as mean ± SD. Normality of data distribution was assessed *via* Shapiro-Wilk tests. Participant age and height were compared for GROUP (HL; LL + BFR; CON) *via* a one-way analysis of variance (ANOVA). Body mass and body mass index (BMI) were analyzed *via* mixed-model ANOVA comparing for GROUP × SESSION (Session 1; Session 21). LOP and training restriction pressures for LL + BFR were compared between sessions *via* paired t-tests. 1-RM strength data were analyzed with a mixed-model ANOVA comparing for GROUP (HL; LL + BFR; CON) and TIME (PRE; MID; POST). Muscle CSA data were analyzed *via* mixed-model ANOVA comparing for GROUP × TIME (PRE; POST).

The ratio of phosphorylated forms to total content (phospho/total) of analyzed intramuscular proteins was also expressed as fold change and analyzed by two-way ANOVA within sessions (Session 1 and Session 21, separately) comparing for GROUP × TIME [Pre-exercise (Pre); immediately post-exercise (0 h); 2 h post-exercise (2 h); 24 h post-exercise (24 h)]. A separate two-way ANOVA was performed within individual groups comparing for SESSION × TIME.

For all significant main effects or interactions within ANOVAs, specific differences were further examined using Tukey-Kramer *post-hoc* tests. For all statistical tests, the significance level was set to *p* < 0.05. All analyses were conducted using Stata/SE 14 (StataCorp LLC, Texas, United States).

## Results

Anthropometric measures were not significantly different between groups (Table 1; *p* > 0.05). Body mass and BMI were both significantly greater in CON during Session 21 compared with Session 1 (both measures *p* < 0.001). For LL + BFR, LOP and training restriction pressure measurements were not different between Session 1 and Session 11 (*p* = 0.32).

### Muscle Strength

**Figure 3:**
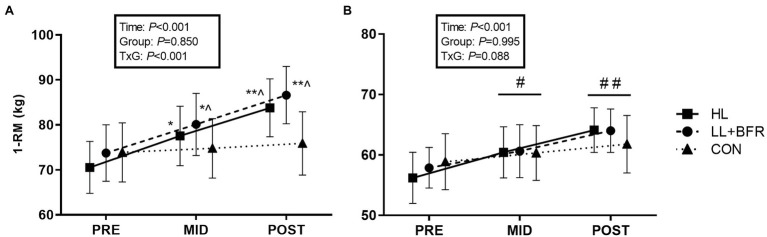
One-repetition maximum (1-RM) strength for bilateral knee extension **(A)** and knee flexion exercises **(B)** in heavy-load resistance training (HL), blood flow restriction training (LL + BFR), and non-training control (CON) groups. Data are mean ± SEM. * Different from PRE (*p* < 0.001); ** different from PRE and MID (*p* < 0.001); ^ different from CON (*p* < 0.01); # different from PRE (TIME main effect; *p* < 0.01); and ## different from PRE and MID (TIME main effect; *p* < 0.01).

Mixed-model ANOVA indicated a GROUP × TIME interaction for knee extension 1-RM strength (Figure 3A; *p* < 0.05). Post-hoc analysis revealed that strength at MID increased significantly in HL (10%) and LL + BFR (9%) when compared to PRE (*p* < 0.001). This was greater at MID in LL + BFR when compared to CON (1%; *p* = 0.04). At POST, knee extension strength increased in HL (19%) and LL + BFR (19%; *p* < 0.001), but not CON (2%; *p* > 0.05). This increase was greater in both HL and LL + BFR compared with CON (*p* < 0.01).

For knee flexion 1-RM strength, there was a main effect for TIME such that strength increased from PRE to MID for ALL groups (Figure 3B; *p* < 0.01), and again at POST (HL = 16%, LL + BFR = 11%, CON = 5%; *p* < 0.01). There was no GROUP main effect or interaction.

### Muscle Cross-Sectional Area

There was no GROUP × TIME interaction (*p* > 0.05) or GROUP main effect (*p* > 0.05) for any muscle CSA measure. A main effect for TIME occurred for total muscle CSA at 25 and 50% femur length, and knee flexor CSA (25% length) indicating an increase at POST for all groups (*p* < 0.01; Table 2). There was no interaction or main effects for knee extensor muscle CSA at 25% femur length (*p* > 0.05).

**Table 2:** Muscle cross-sectional area (CSA) in heavy-load resistance training (HL), blood flow restriction training (LL + BFR), and non-training control (CON) groups.

Cross-section	Site	Group	PRE	POST	Change (%)
25% femur length	Total muscle CSA (cm^2^)	HL	85.9 ± 11.2	87.7 ± 10.3^#^	2.4 ± 6.0
		LL + BFR	84.1 ± 11.4	87.5 ± 10.7^#^	4.2 ± 2.7
		CON	87.5 ± 20.3	89.0 ± 20.1^#^	1.9 ± 2.4
	Knee extensor muscle CSA (cm^2^)	HL	48.5 ± 8.2	48.7 ± 7.1	0.8 ± 5.3
		LL + BFR	46.6 ± 7.6	47.9 ± 7.9	3.0 ± 4.1
		CON	49.2 ± 12.6	49.9 ± 12.9	1.4 ± 3.4
	Knee flexor muscle CSA (cm^2^)	HL	25.9 ± 3.2	27.5 ± 3.6^#^	6.0 ± 5.3
		LL + BFR	25.9 ± 5.2	27.3 ± 4.6^#^	5.8 ± 4.8
		CON	26.7 ± 6.9	27.1 ± 6.7^#^	1.8 ± 3.2
50% femur length	Total muscle CSA (cm^2^)	HL	145.8 ± 17.1	148.9 ± 16.7^#^	2.2 ± 2.9
		LL + BFR	147.6 ± 24.6	151.7 ± 23.7^#^	3.0 ± 3.0
		CON	139.6 ± 33.4	141.8 ± 35.2^#^	2.1 ± 2.7

Data are mean ± SD.

# Main effect for TIME (*p* < 0.01).

### Growth Marker Protein Expression

**Figure 4:**
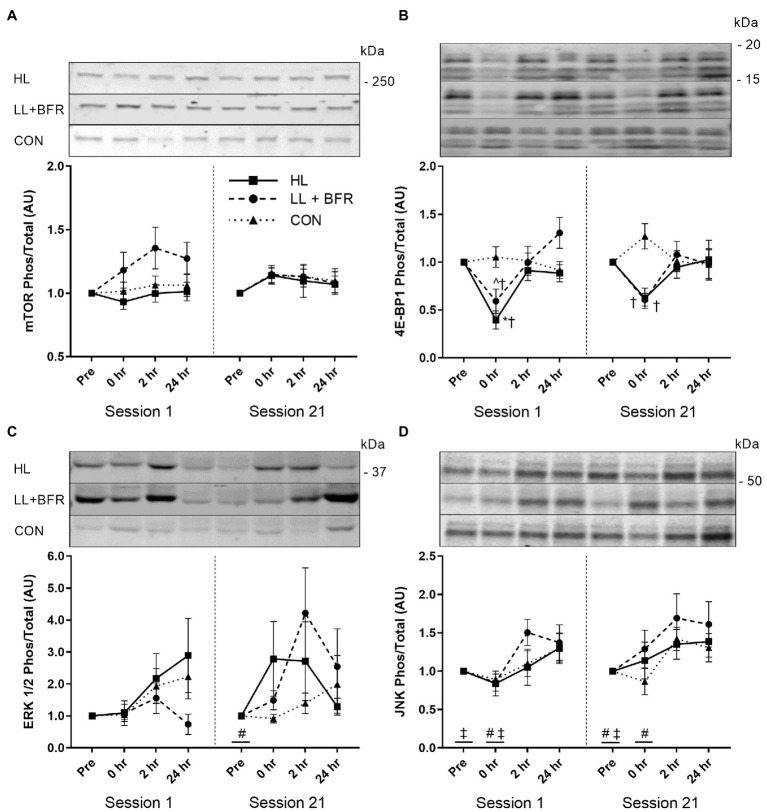
Ratio of phosphorylated to total mTOR (Ser2448; **A**), 4E-BP1 (Thr37/46; **B**), ERK 1/2 (Thr202/Tyr204; **C**), and JNK (Thr183/Tyr185; **D**) sampled pre-exercise (Pre), immediately following (0 h), 2 h following (2 h), and 24 h following (24 h) knee extension and flexion exercise in heavy-load resistance training (HL), blood flow restriction training (LL + BFR), and non-training control (CON) groups during the first (Session 1) and last (Session 21) training sessions of a program. Representative blots also shown. Data are mean ± SEM in arbitrary units (AU; fold change). * Different from all other time points (*p* < 0.05); † different from CON (*p* < 0.05); ^ different from 24 h (*p* < 0.01); # different from 2 h (TIME main effect; *p* < 0.05); and ‡ different from 24 h (TIME main effect; *p* < 0.05). mTOR, mammalian target of rapamycin; 4E-BP1, 4E-binding protein 1; ERK, extracellular signal-regulated kinase; and JNK, c-Jun N-terminal kinase.

No interactions, GROUP, TIME, or SESSION main effects, were identified for mTOR (Ser2448) phosphorylation (expressed as fold change) by two-way ANOVAs (Figure 4A; *p* > 0.05).

For 4E-BP1 (Thr37/46) phosphorylation during both Session 1 and Session 21, two-way ANOVAs indicated GROUP × TIME interactions (Figure 4B; *p* < 0.05). Post-hoc testing identified that in Session 1, 4E-BP1 (Thr37/46) phosphorylation at 0 h (immediately post-exercise) reduced in HL (0.4 ± 0.3 AU) compared with Pre (*p* < 0.001). This was also lower than for 2 h (0.9 ± 0.3 AU; *p* = 0.002) and 24 h (0.9 ± 0.2 AU; *p* = 0.026). 4E-BP1 (Thr37/46) phosphorylation at 0 h for LL + BFR (0.6 ± 0.3 AU) did not reduce compared with Pre (*p* = 0.062), though was lower than 24 h (1.3 ± 0.4 AU; *p* < 0.001). HL and LL + BFR were both lower than CON at 0 h (1.0 ± 0.3 AU; *p* < 0.05). In Session 21, there was no significant change in 4E-BP1 (Thr37/46) phosphorylation for any group. Although, 4E-BP1 (Thr37/46) phosphorylation at 0 h for both HL and LL + BFR was lower than for CON (*p* < 0.05). No SESSION × TIME interaction, SESSION or TIME main effects were identified *via* two-way ANOVA between Session 1 and 21 (*p* > 0.05).

For ERK 1/2 (Thr202/Tyr204) phosphorylation during Session 1, there was no GROUP × TIME interaction or main effects (Figure 4C; *p* > 0.05). Two-way ANOVA identified a TIME main effect within Session 21. Subsequent post-hoc testing revealed Pre was lower overall compared with 2 h (*p* = 0.006). There were no GROUP main effects or interactions for Session 21, and two-way ANOVA between sessions did not identify any SESSION × TIME interaction, SESSION, or TIME main effects (*p* > 0.05).

Two-way ANOVAs for JNK (Thr183/Tyr185) phosphorylation identified TIME main effects for both Session 1 and Session 21 (Figure 4D; *p* < 0.05). Post-hoc analyses revealed that during Session 1, Pre was lower compared with 24 h (*p* = 0.029), and 0 h was lower than 2 h (*p* = 0.014). In Session 21, *post-hoc* tests indicated JNK (Thr183/Tyr185) phosphorylation was lower at Pre compared with 2 h (*p* = 0.007) and 24 h (*p* = 0.019), and was lower at 0 h compared with 2 h (*p* = 0.033). No GROUP main effects or interactions occurred, and there was no SESSION × TIME interaction, SESSION, or TIME main effects identified *via* two-way ANOVA between sessions (*p* > 0.05).

## Discussion

This study aimed to measure and compare the effect of 7 weeks of BFRT and moderately heavy-load resistance training on skeletal muscle adaptations. A non-exercising control group was also included to control for the effect of time. Several key observations were made. Firstly, BFRT and HLRT increased knee extension strength to a similar extent, independently of a meaningful concurrent increase to muscle CSA. This indicates strength adaptations to both resistance training methods were driven by a neuromuscular response. In addition, potential intramuscular mechanisms for protein expression following BFRT appeared similar to those for HLRT, despite differences in primary anabolic stimuli (e.g., training load and metabolic stress).

### Muscle Strength and Cross-Sectional Area

The magnitude of strength adaptation following BFRT has historically been variable due to differences in methodology, though the increase in bilateral knee extension strength observed in the present study following the LL + BFR intervention (19%) is supported by previous literature (Karabulut et al., 2009; Cook et al., 2017, 2018). Knee extension 1-RM strength (and knee flexion strength) also increased similarly to that for HL. This indicates the benefit of BFRT for healthy untrained males and supports its potential as a low mechanical stress alternative to HLRT to induce muscle adaptations for low physical functioning populations. This is particularly applicable for these populations given that safe isolated joint exercises were prescribed, and pneumatic resistance machinery was utilized, which appears to be gaining popularity as a possible training method for older adults and for musculoskeletal rehabilitation. Knee extension 1-RM strength adaptation can be pronounced during BFRT, although the similar adaptation between resistance training groups was somewhat surprising given that improvements to BFRT can sometimes be half that for HLRT (Martín-Hernández et al., 2013; Cook et al., 2017, 2018). It is still unclear if BFRT is as effective as HLRT for strength adaptation (Lixandrão et al., 2018; Grønfeldt et al., 2020), though benefits to BFRT compared with HLRT are likely dependent on factors, such as the training population, exercise selection, and domains of strength (i.e., isometric and isotonic).

Bilateral knee flexion 1-RM strength increased similarly between all groups (TIME main effect). The magnitude of change was lowest for the passive control group (5%), although the main effect for TIME suggests a learning effect on strength adaptation for all groups. The increase in bilateral knee flexion 1-RM strength for LL + BFR (11%; 8 kg) is similar to a previous investigation in women (Seo et al., 2016), though was somewhat less than a previous report from our laboratory with a similar population and training protocol (18%; May et al., 2018). This may have been influenced due to differences in knee flexion exercises (i.e., prone knee flexion in the present study vs. seated previously) as the magnitude of hamstrings muscle adaptation appears influenced long muscle lengths (Maeo et al., 2021). Although, knee extension 1-RM strength was also lower than we have previously shown despite similar baseline strength. Overall, this may be attributable to differences in utilized exercise machinery for resistance exercises; pneumatic resistance machinery was used in the present study for training and testing versus traditional plate-loaded machinery for our previous investigation. There is little information available on comparable effectiveness of these training methods for muscle adaptation. Although, plate-loaded machinery utilizing traditional resistance training (non-BFRT) appears to elicit greater muscle power adaptation in older adults (Balachandran et al., 2017) and induces greater peripheral and central fatigue for inducing strength/hypertrophy adaptation (Peltonen et al., 2013). It is currently unclear how utilizing different exercise machinery would influence results for BFRT.

Training protocols in the current study did not induce meaningful skeletal muscle hypertrophy. The increase in muscle CSA was similar for all groups including CON (TIME main effect; Table 2). Across the two measurement slices (25 and 50% femur length), BFRT increased total muscle CSA on average by 3.6%, HLRT by 2.3%, and passive rest by 2%. Interestingly, the majority of this minor change occurred in the knee flexors, rather than the knee extensors where total muscle mass is greatest and largest strength adaptations were observed. This indicates that the contribution of hypertrophy to strength adaptation following BFRT was negligible and suggests that muscle groups may have different predispositions to hypertrophy in response to BFRT. Distribution of muscle fiber types is variable between different skeletal muscles (Elder et al., 1982). The hamstrings are predominantly composed of fast-twitch fibers (Evangelidis et al., 2017), and BFRT has been suggested to have enhanced activation of fast-twitch fibers, despite the low training load (Pearson and Hussain, 2015). Therefore, this fiber type distribution may be a contributing factor to the preferential hypertrophy of the knee flexors in LL + BFR within this study. It should also be noted that in CON only, body weight and BMI significantly increased from Session 1 to Session 21 (Table 1). Potentially, inactivity in CON participants during over the 7-week training period resulted in accumulation of fat mass. Increases to fat mass and muscle fat free mass can occur concurrently in untrained populations (Bray et al., 2012), potentially due to a requirement for greater muscle mass to support movement of an overall heavier body mass.

The failure of training interventions in this study to induce meaningful skeletal muscle hypertrophy despite significant strength improvement provides further evidence that BFRT, like HLRT, increases strength mostly *via* neurological mechanisms. This was somewhat expected as strength adaptations following BFRT can outweigh hypertrophy (Laurentino et al., 2012; Lixandrão et al., 2015). However, the exact neurological mechanisms by which skeletal muscle strength adaptations occur following BFRT remain unclear. To illustrate, strength adaptations have been observed following BFRT without increased central activation (Kubo et al., 2006; Cook et al., 2018), nerve conduction velocity (Clark et al., 2011), spinal excitability (Takarada et al., 2000b), or peripheral neuromuscular adaptation (*via* electrically evoked torque; Cook et al., 2018). Although, our laboratory has previously found increased motor-evoked potential following BFRT (Brandner et al., 2015). In addition, metabolic stress/accumulation has been suggested to drive BFRT-induced muscle hypertrophy (Pearson and Hussain, 2015). However, this may also be associated with neurological mechanisms for strength adaptation due to a suggested capacity for metabolic accumulation to augment muscle activation (Dankel et al., 2017).

### Growth Marker Activation and Content

Post-exercise mTOR (Ser2448) phosphorylation was similar between acute HLRT and BFRT, and did not change at any time point. Therefore, different exposures to primary anabolic stimuli (i.e., mechanical tension and metabolic stress) *via* HLRT and BFRT had little influence on local mTOR activation. Unchanged post-exercise mTOR (Ser2448) phosphorylation supports some previous evidence for BFRT (Fujita et al., 2007; Wernbom et al., 2013). Although mTOR (Ser2448) activation may increase 1–2 h post-HLRT (Dreyer et al., 2006, 2008; Camera et al., 2010), which we did not observe. As such, the present intramuscular responses support the similar low rates of muscle hypertrophy. However, post-exercise mTOR (Ser2448) phosphorylation has also previously increased at 3 h post-exercise, and not during 1–2 h post-exercise (Fry et al., 2010; Gundermann et al., 2012), suggesting that the chosen biopsy intervals may not have been as optimal for this specific marker as for the others assessed.

4E-BP1 (Thr37/46) phosphorylation is often reduced during the catabolic state induced by resistance exercise (Dreyer et al., 2006, 2008; Deldicque et al., 2008). This reduction immediately post-exercise for HL was consistent with prior studies (Dreyer et al., 2006, 2008, 2010; Camera et al., 2010). Reduced 4E-BP1 (Thr37/46) phosphorylation during exercise is thought to stimulate tissue remodeling following exercise completion (Kraemer and Ratamess, 2005). As such, this downstream factor of the mTOR pathway likely has an anabolic effect following HLRT. 4E-BP1 (Thr37/46) phosphorylation in Session 1 for LL + BFR also followed the trend of the literature as it did not reduce immediately post-exercise (Fry et al., 2010; Gundermann et al., 2012). There are numerous mechanisms for activation of the mTOR pathway influenced by mechanical tension, including insulin-like growth factor-1 signaling and stretch-activated calcium ion channels and integrins (Philp et al., 2011). Potentially, the unchanged 4E-BP1 (Thr37/46) phosphorylation for LL + BFR may be related to insufficient mechanical tension during BFRT. However, it should be noted that 4E-BP1 (Thr37/46) phosphorylation immediately post-exercise in LL + BFR was significantly lower than CON and similar to HL. As such, an effect of BFRT cannot be discounted.

ERK 1/2 is activated through numerous sources; broadly including growth factors, hormones, cytokines, and integrins (Widegren et al., 2001). HLRT can increase ERK 1/2 (Thr202/Tyr204) phosphorylation immediately post-exercise (Karlsson et al., 2004; Deldicque et al., 2008; Drummond et al., 2008). In contrast, acute BFRT has only trended (*p* < 0.1) toward increasing ERK 1/2 (Thr202/Tyr204) phosphorylation (Gundermann et al., 2012, 2014). Phosphorylation within the present study was similar in all groups as only a main effect for TIME (Session 21) was observed, suggesting the high mechanical loads of HL did not induce optimal ERK 1/2 signaling. Similarities in ERK 1/2 (Thr202/Tyr204) phosphorylation between HL and LL + BFR may be associated with the influence of an anabolic hormonal stimulus of BFRT (Takarada et al., 2000a), or the high variability that was observed in ERK 1/2 (Thr202/Tyr204) activation. In addition, this TIME main effect also reflects an upward trend in CON activation indicating an influence separate from resistance training. Multiple muscle biopsies, when taken from the same site, increase ERK1/2 (and JNK) phosphorylation (Aronson et al., 1998). While our biopsies were taken from different sites, the third and fourth biopsies were taken distally (~2 cm apart) from the first and second biopsies, respectively. It is possible that the localized biopsy damage to the muscle may be somewhat causative for the increased phosphorylation over time (TIME main effects) seen for MAPKs within this study.

JNK is linked to mRNA expression of transcription factors that modulate cell proliferation and DNA repair (Schoenfeld, 2010), and appears to increase in activation with rising muscle force output (Martineau and Gardiner, 2001). Although, the role of JNK in HLRT-induced hypertrophy is not yet established (Schoenfeld, 2016), minor increases to phosphorylation for all groups (TIME main effect) suggest JNK activity was not strongly dependent on the magnitude of mechanical tension/loading. Muscle damage appears to be an alternate stimulus for JNK activation and subsequent DNA repair (Schoenfeld, 2010). Muscle damage is negligible during BFRT (Loenneke et al., 2014), and JNK (Thr183/Tyr185) phosphorylation is much lower in concentric vs. eccentric resistance exercise (Boppart et al., 1999). As such, similarities to JNK activation between groups may have occurred due to low muscle damage induced by both resistance exercise modes during the sampling sessions.

To our knowledge, this is the first study to investigate the change in acute intramuscular growth marker signaling after completion of a BFRT program compared to pre-intervention. Phosphorylation of all chosen growth markers for LL + BFR did not differ between Session 1 and 21. This indicates that the response to exercise is preserved/consistent following a program of BFRT. However, 4E-BP1 (Thr37/46) phosphorylation immediately post-exercise in HL during Session 21 was lower than CON but, unlike Session 1, remained similar to pre-exercise. This is the only study we are aware of in which a traditional resistance training program inhibited the post-exercise reduction in 4E-BP1 (Thr37/46) phosphorylation in skeletal muscle. Dreyer et al. (2006) suggested catabolic activity increases during HLRT because protein synthesis requires ATP that is prioritized for muscle contraction. Resistance training can increase availability of ATP, creatine phosphate, and glycogen within muscle (MacDougall et al., 1977). HL may have increased ATP availability, reducing the requirement for ATP sparing and catabolic activity during exercise. Therefore, the greater phosphorylation of 4E-BP1 (Thr37/46) during Session 21 may suggest that the HLRT program may have reduced the stimulus for tissue remodeling, subsequently resulting in a low rate of muscle hypertrophy. Conversely, though progressive overload was enforced in this study, it is possible that the mechanical stress of HLRT simply reduced as participants became more familiar with the knee flexion and extension exercises.

### Limitations

The observed growth marker protein activation was minor. This can be attributed to minor muscle hypertrophy within the present study and so may not be representative of the intramuscular environment of training protocols with greater physical muscle adaptation. The reported protein activation is also dependent on selection of the vastus lateralis as the chosen site of muscle biopsies. The measured intramuscular activation of growth marker proteins is only a representative snapshot of activity within the knee extensors, where knee extensor muscle CSA remained unchanged at 25% femur length. Furthermore, this was only a preliminary time course comparison of protein growth marker signaling between training groups. Changes to intramuscular signaling may be more clearly observed in future if the Akt pathway was further characterized. For example, post-exercise changes to activation of growth marker proteins within the downstream FoxO pathway were not assessed despite these signaling cascades having a major role in catabolic activity within skeletal muscle (Accili and Arden, 2004).

The minor skeletal muscle hypertrophy for training groups observed in the present study reflects a likely neurological mechanism for strength adaptation during shorter training programs. However, the low magnitude of change was not expected. We identified four participants across training groups that were non-responders across multiple measurement sites (i.e., 25% length, 50% length, flexors, and extensors). As global non-responders to exercise are unlikely to exist but rather benefit from a more individualized response (Pickering and Kiely, 2019), our study protocol was perhaps overly rigid. Exercise prescription may have benefited from greater flexibility in programming variables, such as rest period durations, the applied BFR pressure, and progressive overload.

## Conclusion

This study found that the magnitudes and mechanisms for strength adaptation and intramuscular anabolic activity following a 7-week BFRT program are similar to those for moderately heavy-load resistance training. Minor hypertrophy with large strength adaptation reaffirms the contribution of the neuromuscular system in driving strength adaptation in response to resistance exercise of differing forms. This occurred despite differences in the nature of training methods, such as exercise load or induced metabolic stress, indicating consistent mechanisms by which muscle adapts to resistance-based exercise programs.

## Data Availability Statement

The raw data supporting the conclusions of this article will be made available by the authors, without undue reservation.

## Ethics Statement

The studies involving human participants were reviewed and approved by the Deakin University Human Ethics Advisory Group – Deakin University. The patients/participants provided their written informed consent to participate in this study.

## Author Contributions

AM, SW, PG, and AR conceived and designed the research, wrote, edited, and approved the manuscript. AM conducted the experiments. AM and SW analyzed the data. All authors contributed to the article and approved the submitted version.

## Funding

This research was supported only by local funds made available by the School of Exercise and Nutrition Sciences, Faculty of Health, Deakin University, Victoria, Australia.

## Conflict of Interest

The authors declare that the research was conducted in the absence of any commercial or financial relationships that could be construed as a potential conflict of interest.

## Publisher’s Note

All claims expressed in this article are solely those of the authors and do not necessarily represent those of their affiliated organizations, or those of the publisher, the editors and the reviewers. Any product that may be evaluated in this article, or claim that may be made by its manufacturer, is not guaranteed or endorsed by the publisher.
