# Supplementary material for: Muscle Adaptations to Heavy-Load and Blood Flow Restriction Resistance Training Methods
Source: Front Physiol. 2022 Feb 3;13:837697. doi: 10.3389/fphys.2022.837697 (PMC8850930; doi:10.3389/fphys.2022.837697)
Supplement: Supplementary file 1 [file Data_Sheet_1.docx]

Supplementary Material

Below shows representative membranes for Western blot analysis. Blots for Phospho-mTOR (Ser2448), Total mTOR, Total S6K1, Total MuRF-1 and Phospho-4E-BP1 (Thr37/46) were analyzed from a single membrane cut at specified weights. As were Phospho-SAPK/JNK (Thr183/Tyr185), Total SAPK/JNK, Phospho-ERK 1/2 (Thr202/Tyr204), Total ERK 1/2 and Total 4E-BP1, on a separate membrane.


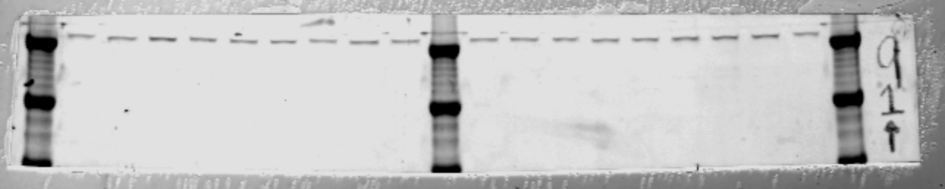


**Supplementary Figure 1.** Phospho-mTOR (Ser2448); 1:1,000 antibody dilution (Rabbit); Cell Signalling Technology. Membrane shows 100-250 kDa.


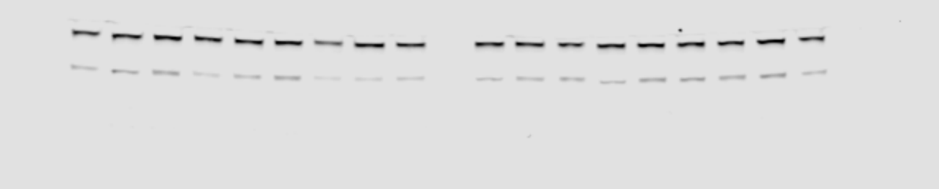


**Supplementary Figure 2.** Total mTOR; 1:1,000 antibody dilution (Mouse); Cell Signalling Technology. Membrane shows 100-250 kDa.


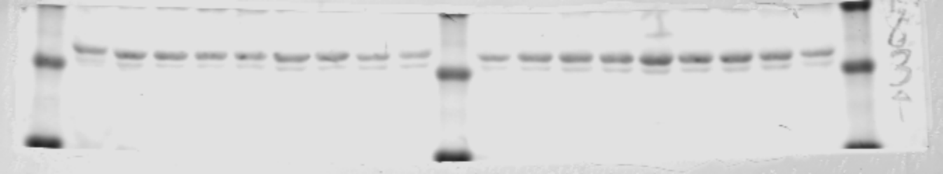


**Supplementary Figure 3.** Total S6K1; 1:1,000 antibody dilution (Rabbit); Cell Signalling Technology. Membrane shows 50-100 kDa.


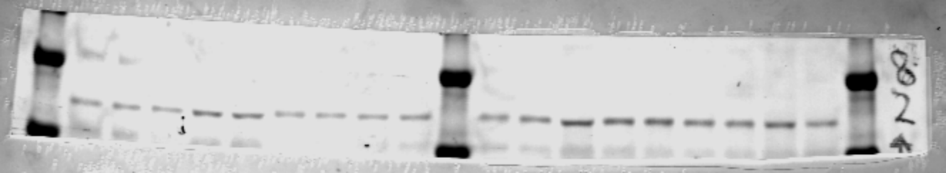


**Supplementary Figure 4.** Total MuRF-1; 1:1,000 antibody dilution (Rabbit); Taylor Bio-Medical. Membrane shows 25-50 kDa.


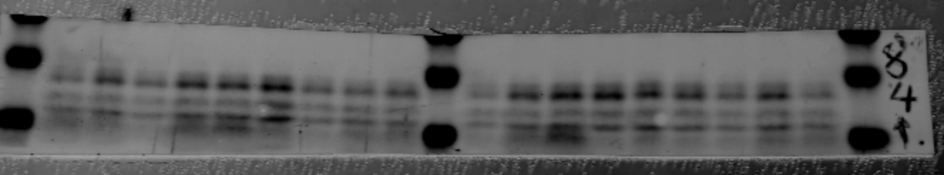


**Supplementary Figure 5.** Phospho-4E-BP1 (Thr37/46); 1:1,000 antibody dilution (Rabbit); Cell Signalling Technology. Membrane shows 15-25 kDa.


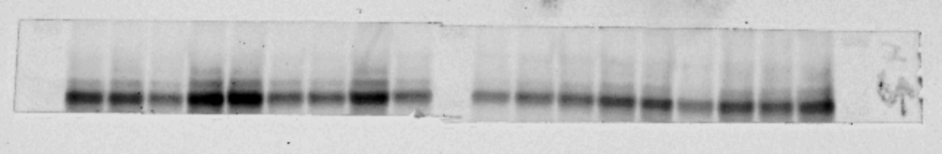


**Supplementary Figure 6.** Phospho-SAPK/JNK (Thr183/Tyr185); 1:1,000 antibody dilution (Mouse); Cell Signalling Technology. Membrane shows 50-75 kDa.


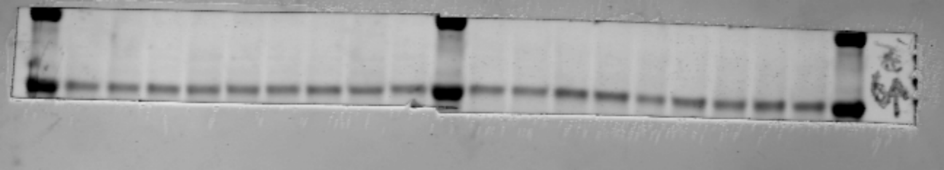


**Supplementary Figure 7.** Total SAPK/JNK; 1:1,000 antibody dilution (Rabbit); Cell Signalling Technology. Membrane shows 50-75 kDa.


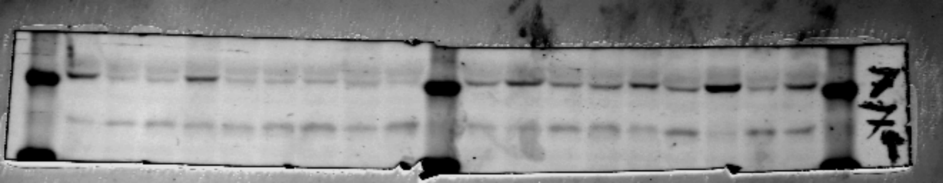


**Supplementary Figure 8.** Phospho-ERK 1/2 (Thr202/Tyr204); 1:2,000 antibody dilution (Rabbit); Cell Signalling Technology. Membrane shows 25-45 kDa.


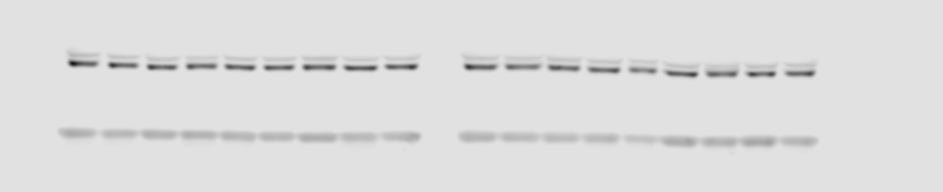


**Supplementary Figure 9.** Total ERK 1/2; 1:1,000 antibody dilution (Mouse); Cell Signalling Technology. Membrane shows 25-45 kDa.


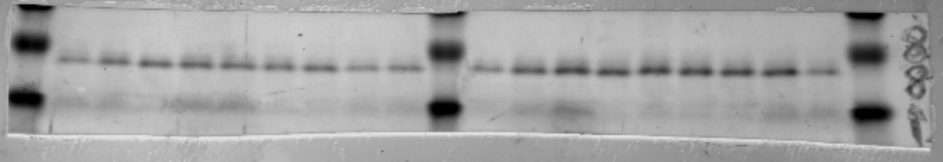


**Supplementary Figure 10.** Total 4E-BP1; 1:500 antibody dilution (Rabbit); Cell Signalling Technology. Membrane shows 15-25 kDa.

**Supplementary Table 1**: Pre-exercise protein growth marker content and activation in training Session 1 and Session 21 in heavy-load resistance training (HL), blood flow restriction training (LL+BFR), and non-training control (CON) groups.

| **Protein** | **Form** | **Group** | **Session 1** | **Session 21** |
| --- | --- | --- | --- | --- |
| **mTOR** | mTOR (Ser2448)/Total | HL | 1.6 ± 1.1 | 1.4 ± 0.8 |
|  |  | LL+BFR | 1.5 ± 0.7 | 1.6 ± 0.6 |
|  |  | CON | 1.0 ± 0.3 | 1.0 ±0.4 |
|  | Total | HL | 3.4 ± 1.4 | 4.0 ± 1.6 |
|  |  | LI-BFR | 3.5 ± 1.6 | 4.0 ± 2.5 |
|  |  | CON | 4.3 ± 1.6 | 4.2 ± 1.5 |
| **S6K1** | Total | HL | 2.6 ± 0.9 | 2.6 ± 0.7 |
|  |  | LL+BFR | 3.2 ± 1.0 | 3.2 ± 0.9 |
|  |  | CON | 3.1 ± 0.9 | 3.5 ± 1.2 |
| **4E-BP1** | 4E-BP1 (Thr37/46)/Total | HL | 0.9 ± 0.6 | 0.9 ± 0.4 |
|  |  | LL+BFR | 0.5 ± 0.2 | 0.7 ± 0.4 |
|  |  | CON | 0.8 ± 0.4 | 0.8 ± 0.5 |
|  | Total | HL | 2.2 ± 1.9 | 1.7 ± 0.6 |
|  |  | LL+BFR | 2.8 ± 2.0 | 2.3 ± 1.3 |
|  |  | CON | 2.2 ± 1.1 | 2.1 ± 1.2 |
| **ERK 1/2** | ERK 1/2 (Thr202/Tyr204) /Total | HL | 1.1 ± 0.6 | 1.4 ± 1.6 |
|  |  | LL+BFR | 2.4 ± 1.8 | 1.0 ± 0.9 |
|  |  | CON | 1.0 ± 0.5 | 0.7 ± 0.4 |
|  | Total | HL | 1.5 ± 0.4 | 1.7 ± 0.6 |
|  |  | LL+BFR | 1.7 ± 0.4 | 2.0 ± 0.6 |
|  |  | CON | 1.6 ± 0.2 | 1.5 ± 0.3 |
| **JNK** | JNK (Thr183/Tyr185)/Total | HL | 1.0 ± 0.9 | 0.7 ± 0.3 |
|  |  | LL+BFR | 0.7 ± 0.3 | 0.8 ± 0.5 |
|  |  | CON | 1.0 ± 0.5 | 0.8 ± 0.2 |
|  | Total | HL | 3.1 ± 1.0 | 3.4 ± 1.0 |
|  |  | LL+BFR | 3.2 ± 1.0 | 3.5 ± 1.3 |
|  |  | CON | 2.7 ± 0.9 | 2.8 ± 0.4 |
| **MuRF-1** | Total | HL | 1.7 ± 0.5 | 1.6 ± 0.3 |
|  |  | LL+BFR | 2.1 ± 0.5 | 2.0 ± 0.5 |
|  |  | CON | 2.0 ± 0.5 | 2.2 ± 0.5 |

Data are mean ± SD in arbitrary units (AU). Pre-exercise intramuscular content and activation of growth marker proteins within Session 1 and Session 21 was analyzed via mixed-model ANOVA comparing for GROUP × SESSION (Session 1; Session 21; supplementary material). No changes or group differences observed. mTOR, mammalian target of rapamycin; S6K1, ribosomal S6 kinase 1; 4E-BP1, 4E-binding protein 1; ERK 1/2, extracellular signal-regulated kinase 1/2; JNK, c-Jun NH2-terminal kinase; MuRF-1, muscle RING finger protein-1.
